# Registration of Foreign Diplomas in England

**Published:** 1893-08

**Authors:** 


### EDITORIAL.

### Registration of Foreign Diplomas in England.

In the July number of the DENTAL REGISTER is published a report by the Education Committee to the General Medical Council, and the action of the council in reference to the registration of foreign diplomas. The report presents the following:

"GENERAL MEDICAL COUNCIL OFFICE, 299 OXFORD STREET,
LONDON, W., MAY 31, 1893.

"*Sir*:—By order of the General Medical Council I send you herewith a copy of a report presented to the council by its Education Committee whereon the council at its meeting on the 29th inst. passed the following resolution:

"That the recognition of the certificates of the degrees of Doctor of Dental Medicine of the University of Harvard, and Doctor of Dental Surgery of the University of Michigan by the General Council be suspended until further notice, and that the REGISTRAR be instructed to refuse registration of such certificates.

I am, Sir, Yours Faithfully,

W. J. C. MILLER, Registrar."

"The Sec'y of the Den. Dep't of the Uni'y of Michigan."

Now that the Dentists' Act of 1879 gives full discretionary power in this matter t› the General Medical Council the Ninth and Tenth Sections of the Act, here given, clearly shows:

"SEC. IX. Where a person who is not a British subject, or who has practiced for more than ten years elsewhere than in the United Kingdom, or in the case of persons practicing in the United Kingdom at the time of the passage of this Act for not less than ten years either in the United Kingdom or elsewhere, shows that he obtained some recognized certificate [as hereinafter defined] granted in a foreign country, and that he is of good character, and either continues to hold such certificate, or has not been deprived thereof for any cause which disqualified him for being registered under this Act, such person shall upon payment of the registration fee be entitled, without examination in the United Kingdom, to be registered as a foreign dentist in the Dentists' Register.

"SEC. X. The certificate granted in a British possession or in a foreign country, which is to be deemed such a recognized certificate as is required for the purposes of this Act, shall be such certificate, diploma, membership, degree, license, letters, testimonials or other title, status or document as may be recognized for the time being by the General Council as entitling the holder thereof to practice dentistry, or dental surgery in such possession or country, and as furnishing sufficient guarantees of the possession of the requisite knowledge and skill for the efficient practice of dentistry or dental surgery.

"If a person is refused registration as a colonial dentist, or as a foreign dentist, the general registrar shall, if required by him, state in writing the reason for such refusal, and if such reason be that the certificate held or obtained by him is not such a recognized certificate as above defined, such person may appeal to the Privy Council, and the Privy Council, after hearing the General Council, may dismiss the appeal, or may order the General Council to recognize such certificate, and such order shall be duly obeyed."

The ninth section was the authority for the recognition which was extended to graduates of Harvard and Michigan, and, furthermore, this act of recognition was based upon a supposed higher standard of requirements by these two institutions than had been established by similar schools in the United States. This action of the council was had early in the year 1879. The Executive Committee of the Medical Council at its regular meeting, held on the 5th of March, in their report used the following language, or made the following recommendation: "With regard to fifteen foreign and colonial universities and colleges who grant dental diplomas the executive committee recommend that the dental degree granted by the Harvard University and by the University of Michigan be recognized by the council as qualifying for registration. Of the other institutions the committee feel that further information is needed respecting the courses of study and the examinations before they can recommend the council to recognize the certificates granted by them." This recommendation was approved by the council.

Now, certainly, it cannot be affirmed of our English brethren that when they start out on a line of investigation of this sort that they will be satisfied with half-way work, nor are likely to be imposed upon by defective evidence. This recognition was a voluntary act. It was accorded without solicitation or suggestion by the faculties of Harvard and Michigan, or even a knowledge of the proposition involving the recognition until after it was accomplished.

In discussing this action of the Medical Council the *Monthly Review of Dental Surgery*, for April, 1879, says: "It would be obviously unjust to exact a higher, or to accept a lower education from a foreigner who proposes to settle in this country than that required of our own practitioners. Acting under this indisputable rule it is distinctly shown in the registrar's schedule that the council could not accept for registration more than two of the foreign qualifications, namely, the dental diplomas of the Universities of Harvard and Michigan. In each of these schools it is required that three years should be devoted to professional studies, whether for dental or medical diploma, and the subjects common to each, are in each case, to be attended at a recognized medical school."

This recognition, coming as it did, was a matter of gratification to the two institutions referred to.

In the recent action by which this recognition "was suspended for the time being" the Medical Council acted in entire accord with the authority vested in it by the Dentists' Act, as will be seen by noting Section X. Of course, the recognition could have been suspended or withdrawn without any reasons being given for it, but it was thought best to state some grounds for the action, and the first point given is: "inferior preliminary or entrance qualification required by Harvard and Michigan as compared with those of the English colleges," and in order that every one may see and judge for himself the published proceedings of the council give, in parallel columns, a list of the subjects of each upon which the entrance examination is based. In regard to which it may be said that an examination embracing the subjects in either of these lists may be good or indifferent according to the course pursued. If the examination is conducted in good faith and brings out fully the attainments of a student, and he proves to be well up in each of the branches here named he will have a fairly good preparation for entering upon the study of his professional course. In regard to the preliminary examination for entrance to Michigan, and we presume the same is true of Harvard, the preliminary requirements have been raised during the last ten years, so that now a much larger range of subjects is embraced, and a more thorough examination made than at that time. Almost every year something has been added in this respect. These examinations are not conducted by the dental faculty, but the applicants for dentistry, medicine, and pharmacy are examined together by a Board of Examiners appointed by the university, and the examination made without the knowledge of the Board of Examiners as to the assignment of the students, so the statement that; "The candidates are examined at the colleges that are waiting to receive them as pupils, or what is more extraordinary, private indviduals are empowered to examine candidates and to certify to their fitness to become students at the university," needs to be revised. Students are not examined at the department which they are to enter, nor are parties outside, though they are designated to have applicants write answers to questions, empowered to decide, or certify to their fitness. The answers are returned to the committee of examination at the university, and by that committee have their value determined. The reason for designating private individuals at distant points to do this work is for the accommodation of the students, that they may, if they choose, have some knowledge of the probability of their entrance before making a long and expensive journey.

The medical branches embraced in the curriculum of both these schools is quite as extensive as the time alloted will permit, and in the main, this work is done by the dental students in and with the classes of medical students; some special work on most of these branches is done with and for the dental student outside of the medical classes.

We are quite in accord with the intimation put forth in this report, that three terms of nine months each is too short a time for the average student to master all the subjects of the curriculum, as presented by both Harvard and Michigan. But to this it may be replied, that quite a number of the students at Michigan, at least, take four years, and it is now contemplated at an early date to extend the requirements to that time. Those who are thorough students, industrious, persevering, economical of time, will in a very good degree master all the subjects of this curriculum, but for others possessing these qualities in a less degree it is very laborious, and some cannot accomplish it at all in three years. Quite a goodly number of the students in both these institutions have a year more or less of private pupilage. Sometimes this is a benefit, in other cases of no value, and in other cases a positive injury ; and may it not be that our English brethren have experienced something of this kind. A studentship, whether apprenticed or not, whether for one year or three, is valuable only in proportion to the ability and faithfulness of the preceptor as a teacher, and the industry and natural ability of the student.

In this report a comparison is made between the fees charged in the English and American schools, and the intimation is made that, of course, where low fees are charged the instruction must be of inferior character. This, we think, is wholly an unwarranted inference ; the fees charged have nothing to do with the work of the teachers nor with that of the students. The control of the finances of the Michigan school is wholly in the hands of the University authorities. The University is established and sustained by the State ; it was organized originally to practically give free education to those admitted to its portals. If any State or country chooses to do this without permitting this fact in any wise to influence its legitimate work, it is its province and right to do so, whoever may criticise. The financial question is one that need not enter into this question in any particular. The mere fact that the fees for a course of instruction in England amount to one hundred and twenty-two pounds while in the Michigan school the fees are only thirty-seven pounds, fourteen shillings, will hardly be accepted by anyone as a gauge of the comparative value of the instruction given in the two cases.

In this report quite a point is made upon the fact that the examination of the students for graduation is made by the teachers—members of the faculty. Well! what of it? Is it possible that any independent examiners who have known nothing of the progress of the students, know little or nothing of their natural abilities, can determine as accurately as faithful teachers, who labor for three years in giving instruction to the student, and who must know and recognize all that the student learns through this period; no faithful and well qualified teacher would presume to make his decision as to the qualifications of a student upon the final examination, he has formed his decision in regard to the student from the first day he begins to teach him, on to the conclusion of his work with him. Altogether too much importance is usually attached to final examinations in all educational departments, and it would be well if they were altogether abandoned. If a teacher in his didactic work, in the recitations, in the quizzes, in the notes of the students, in the papers and essays which he should be frequently required to write, is not able to ascertain the qualifications of the student without a final examination of sixty to ninety minutes, his work has been either faulty or he has been seriously negligent.

A letter from Mr. Morton Smale, which is embodied in the report, presents a thought or two worthy of note. He says, "When the two American Colleges were admitted to our home *Register*, it was certainly never contemplated that they were to be able to send to us foreigners of any or all nationalities. Yet in so doing they are acting within the rights conceded to them."

"According to this, the two American Colleges, having access to our *Register*, have the whole population of the world as their possible students, and, with their diplomas, are eligible for our home *Register*."

When we take into account that not more than twenty to thirty dentists bearing diplomas from these two institutions have sought to register in England, does it not appear that Mr. Smale is easily alarmed. A far larger number of English dentists are practicing in the United States, and we have not yet learned of any one being alarmed on that account. All that have come, or all that may come, will be heartily welcome here, and especially if they have been well trained in the english schools. It is true, four or five of our States have laws conferring upon their Boards of Examiners the power to examine any and all persons who propose to practice dentistry within their bounds; but this pertains to our own colleges as well as to those of all other countries; but there are more than forty States and territories that are open to the graduates or licensees of all reputable colleges in this or any other country. Is it possible that the few graduates who have gone to Great Britain to practice their profession are likely to revolutionize the practice in that country? We do not believe that they have any such intention or could accomplish it if they had.

Mr. Smale says, further, "Excepting always natives of Great Britain, therefore, these two American Colleges, with a shorter and inferior curriculum, and inferior examination, are very likely to send more foreign applicants for registration than are likely to come from our home schools."

The probability is that if Mr. Smale's knowledge of the condition of things here was equal to his energy when he wrote this letter he would be inclined to make some modification. Indeed, we are rather of the opinion that the letter contains suggestions and statements that should not have been presented in this connection, especially when the reasons given by the Medical Council for the recognition of these schools in 1879 are taken into consideration.

---

In a recent editorial in The International Dental Journal, bearing the caption "Sunshine and Shadow," in which the writer discusses the dangers that attach to the practice of dentistry. In one paragraph is the following statement: "The practice of dentistry is beyond question one of most laborious and destructive to health of all the professions. The vitiated atmosphere, the constrained positions, the nervous strain, the unnoted pressure of the chair upon the body resulting oftentimes in serious lesions, the monotony of practice, all combined render it difficult and unsatisfactory, if the reason be not allowed full supremacy in governing it."

The first statement of this paragraph will not be accepted by all; the question as to whether it is one of the most laborious and destructive to health of all the professions depends upon the manner in which it is followed. The practice of dentistry is less destructive to the health than the practice of general medicine, and much less than the practice of general surgery. The practice of law may be and oftentimes is quite as injurious to the health as the practice of dentistry. There is scarcely any occupation in which men engage where they may not abuse health. In the dental profession will be found as large a proportion of vigorous, healthy, active men as in any of the other professions. It is true that some men injure their health in dental practice—the same is true in any other profession. If the dentist works in a vitiated atmosphere he is responsible for the results. With the present knowledge of the structure of buildings and how to secure good ventilation and a supply of good air, there is no excuse for anyone working in a vitiated atmosphere. In the arrangement of an office, as everywhere else, where persons are to be occupied for a considerable share of the time, it is practicable and imperative that good air be secured. The world is full of it, and if we do not use it it is our own fault.

The faulty and constrained positions are mentioned. It is true that occasionally an unnatural position must be assumed, but it is by no means necessary that a painfully constrained or an injuriously strained position be maintained for any considerable length of time, there is constant opportunity for change of position and the attainment of relief in this way. It is the testimony of many in the profession that they suffer no inconvenience from the attitude or position necessary in performing the ordinary operations of dental practice. Every beginner in dental practice should make it a point to study the various positions he must assume in his work and so regulate these, as he may in almost every instance, so as to work no physical injury to himself; and that habit once formed will remain.

In regard to the nervous strain there will be great variation. This on an average in dental practice is hardly to be compared with that of the physician or surgeon; nor, indeed, with that of the lawyer or minister who are fully occupied in their legitimate work. Long observation upon this point warrants the statement that there is comparatively less of this in dental practice than in many of the other occupations of life.

There ought to be no unnoted pressure of the chair upon the body that would be prejudicial to health, to say nothing of serious lesions. The chair should be so constructed and so adjusted that it will not press upon the body. (Isn't it rather the body that presses upon the chair?) Not only the chair but all the paraphernalia of the operating room should be most perfect in their arrangement, so that, most perfect ease can be attained in their use.

The next two paragraphs following the one above quoted contain excellent suggestions and advice. Further along in the article we find the following statement: "It would be interesting and very instructive if those who have lived longest in the practice of dentistry and maintained a fair share of health to old age could give their experience." Well, this has been done. Many papers have been read before societies on the hygiene of dental practice; on the care of the dentist's health; the best means of conserving this; and many a time full and free discussion has been had upon the subject; and certainly those who have been in the habit of attending dental societies must have heard these things.

It is not necessary for any one to live in the pursuit of any special calling for thirty to fifty years to ascertain that certain practices and modes are injurious to health and others conservative. The laws of hygiene and well-being are so fully elaborated and explicitly stated that no one inclined to give thought and attention in this direction need be in ignorance on the subject. There is a far larger proportion of dentists injured and too often destroyed by practices and habits, with the effects and power of which they are perfectly familiar, than are caused to suffer by any of the things indicated in the quotation first given above; such for example as the use of tobacco, alcoholic drinks, chloral, morphine, &c., &c. No man indulging in any of these things ought to engage in dental practice, and especially so where the use is excessive.

Many good suggestions are made in the article referred to. The length of time for continuous work should be scrupulously regulated. Three or four hours work without intermission is as long as anyone can afford to persistently work. Regularity of habit in all particulars is a very important item for maintaining health. Irregularity in the partaking of meals, in regard to the time and amount is a very important matter. A sufficient amount of sleep taken regularly and with the least possible interruption is one of the powerful factors in maintaining good health. Avoid all pernicious habits and practices, especially those referred to above. Work from five to eight hours per day according to strength. Permit nothing to interfere with the regularity of partaking of food, and secure seven to nine hours sleep in the twenty-four, have fresh air and sunshine as fully as practicable, and so far as health and strength are concerned under ordinarily good circumstances the dentist can work fifty-two weeks in the year, if he wishes to do so and come out at the end physically as well as when he began; or he may take any time he chooses for healthful, energy-giving recreation. Diversity of thought and occupation is an important matter. If the dentist confines himself to his professional work but six to eight hours a day he will have ample time for all such diversion.

It has not infrequently been observed that those who take two or three months for special recreation require some considerable time for recuperation after returning home. We have known a good many cases of this kind, and some of them were dentists occupying by no means an obscure position.

We extend thanks to brother Truman for the article in question as it is looking in a very important direction.

